# Briacavatolides A–C, New Briaranes from the Taiwanese Octocoral *Briareum* *excavatum*

**DOI:** 10.3390/md10051019

**Published:** 2012-05-02

**Authors:** Tsun-Tai Yeh, Shang-Kwei Wang, Chang-Feng Dai, Chang-Yih Duh

**Affiliations:** 1 Department of Marine Biotechnology and Resources, National Sun Yat-sen University, Kaohsiung 804, Taiwan; Email: m985020027@student.nsysu.edu.tw; 2 Department of Microbiology, Kaohsiung Medical University, Kaohsiung 807, Taiwan; 3 Institute of Oceanography, National Taiwan University, Taipei 106, Taiwan; Email: corallab@ntu.edu.tw; 4 Asia-Pacific Ocean Research Center, National Sun Yat-sen University, Kaohsiung 804, Taiwan

**Keywords:** *Briareumexcavatum*, briarane-type diterpenoid, cytotoxicity, anti-HCMV

## Abstract

In order to search for novel bioactive substances from marine organisms, we have investigated the organic extracts of the Taiwanese octocoral *Briareum* *excavatum* collected at Orchid Island. Three new briarane-type diterpenoids, briacavatolides A–C (**1**–**3**) as well as two known briaranes, briaexcavatolide U (**4**) and briaexcavatin L (**5**) were isolated from the acetone extract. The structures of these compounds were elucidated by extensive NMR spectroscopic analysis and physical data. The anti-HCMV (human cytomegalovirus) activity of **1**–**5** and their cytotoxicity against selected cancer cell lines were evaluated.

## 1. Introduction

Briarane-type diterpenoids, a group of diterpenoids having a highly oxidized bicyclo[8.4.0] system with a γ-lactone group are found only in marine organisms and mainly from octocorals [1,2,3]. The compounds of this type are proven to possess various bioactivities such as anti-inflammatory, cytotoxicity, and antibacterial activity [1,2,3]. Octocorals belonging to the genus *Briareum* (family Briareidae) are recognized as rich sources of briarane-type diterpenoids (3,8-cyclized cembranoid) [1,2,3]. The samples for previous studies on the secondary metabolites of the Taiwanese octocoral *B. excavatum* were all collected along the southern coast of Taiwan [1,2,3].

**Figure 1:**
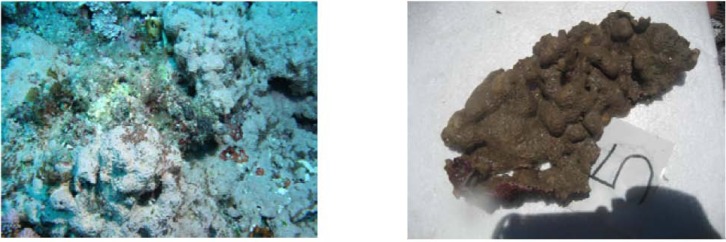
Octocoral *Briareum* *excavatum*.

**Figure 2:**
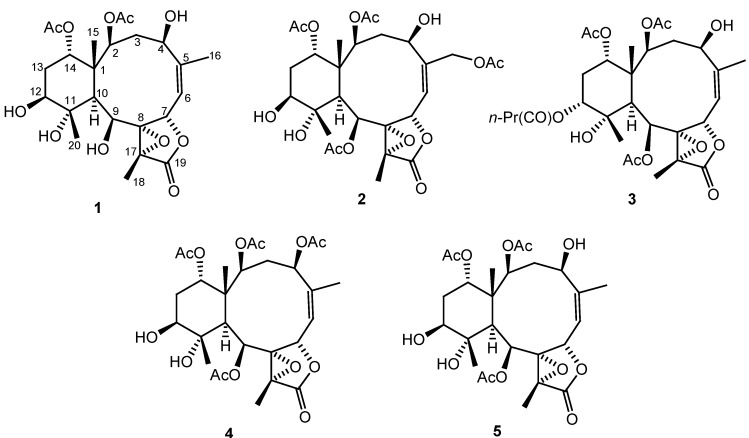
Structures of compounds **1**–**5**.

HCMV is a highly ubiquitous pathogen in human population global prevalence 60~90%. For most healthy people, HCMV remains a long-term subclinical infection, however, in congenital neonates and in immunocompromised patients the virus can cause severe diseases. Of the FDA approved therapeutic agents, ganciclovir, foscarnet, and cidofovir are reported to have adverse effects on bone marrow and the kidneys. The first chemical investigation of *B. excavatum* (Figure 1) collected at Orchid Island off Taiwan during August 2008 afforded three new briarane-type diterpenoids, briacavatolides A–C (**1**–**3**) as well as two known briaranes, briaexcavatolide U (**4**) [4] and briaexcavatin L (**5**) [5] (Figure 2). The anti-HCMV (human cytomegalovirus) activity of **1**–**5 **and their cytotoxicity against selected cell lines were evaluated.

## 2. Results and Discussion

**Figure 3:**
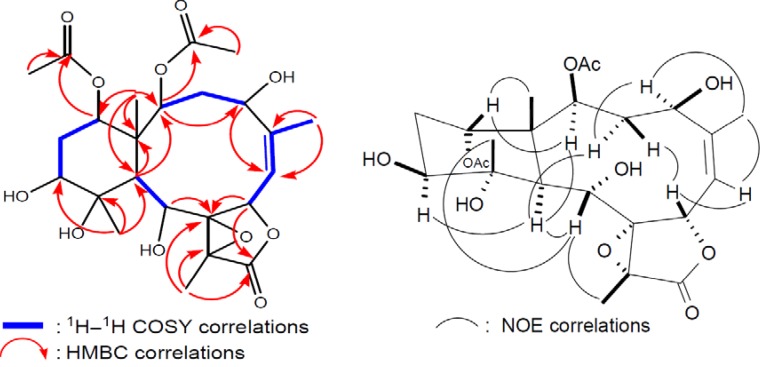
2D NMR correlations of compound **1**.

Briacavatolide A (**1**) was obtained as a white powder. Its HRESIMS and NMR spectroscopic data established a molecular formula of C_24_H_34_O_11_, implying the existence of eight double bond equivalents. The ^13^C NMR spectra showed signs of 24 carbons differentiated by DEPT into six methyls, two methylenes, eight methines and eight quaternary carbons. The ^1^H and ^13^C NMR spectra (Table 1) of **1** indicated the presence of two acetoxyls (*δ*_H_ 1.99, 2.00; *δ*_C_ 21.5, 21.3, 170.4,170.4), a lactone (*δ*_H_ 6.18; *δ*_C_ 173.1), and a trisubstituted olefin (*δ*_H_ 5.27; *δ*_C_ 145.6, 121.8). A tetrasubstituted epoxide containing a methyl substituent was revealed from the signals of two quaternary oxygenated carbons (*δ*_C_ 65.2, 72.0) and a methyl (*δ*_C_ 9.2; *δ*_H_ 1.66, 3H). From the above data, metabolite **1** was found to be a tetracyclic compound. The structure and all of the ^1^H and ^13^C chemical shifts of **1** were determined by the assistance of 2D NMR experiments, including ^1^H-^1^H COSY and HMBC experiments (Figure 3). By explanation of ^1^H−^1^H COSY correlations (Figure 3), it was possible to establish four partial structures of consecutive proton systems extending from H-2 to H-4; H_3_-16 to H-6 through H-5 and H-6; H-9 to H-10; and H-12 to H-14. HMBC correlations (Figure 3) further led to the connectivities of the gross structure. By the above observations, the structure of metabolite **1** could be seen to be very similar to those of a known compound, briaexcavatin L [5] which was previously isolated from the soft coral *B.* *excavatum*. Also, it was found that the acetoxy groups attaching at the C-9 positions in briaexcavatin L were replaced by a hydroxy group by comparing the 1D and 2D NMR data of **1** with those of briaexcavatin L. On the basis of the above finding, and by the NOE correlations observed in the NOESY spectrum of **1** (Figure 3), it was found to be the 9-*O*-deacetyl derivative of briaexcavatin L.

**Figure 4:**
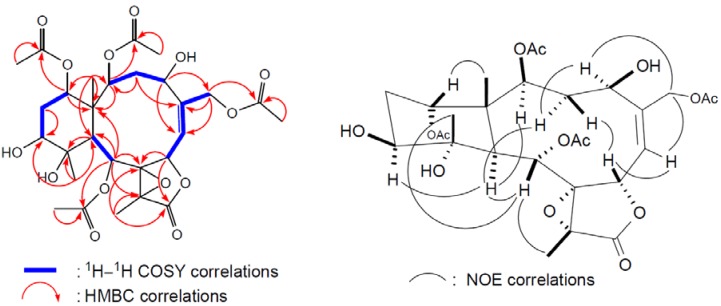
2D NMR correlations of compound **2**.

Briacavatolide B (**2**), had a molecular formula of C_28_H_38_O_14_ as deduced by HRESIMS. The IR spectrum of **2** indicated the presence of hydroxy (3448 cm^−1^), γ-lactone (1777 cm^−1^), and ester (1734 cm^−1^) groups. From the ^13^C NMR data of **2** (Table 1), a trisubstituted olefin (*δ*_C_ 145.3, s, C-5; 125.4, d, CH-6) and five carbonyl resonances (*δ*_C_ 171.5, 170.6, 170.2, 168.3, 4×s, ester carbonyls; 170.2, s, C-19) were observed. The four esters were identified as acetates by the presence of four methyl resonances in the ^1^H NMR spectrum of **2** at *δ*_H_ 2.26 (3H, s), 2.10 (3H, s), 2.00 (3H, s), and 1.98 (3H, s) (Table 1). The planar structure of **2** was determined by 2D NMR experiments (Figure 4). 

**Table 1:** ^1^H and ^13^C NMR data for compounds **1**–**3**.

position	1		2		3	
	*δ*_H_ (*J* in Hz) *^a^*	*δ*_C_ (mult.) *^b^*	*δ*_H_ (*J* in Hz) *^a^*	*δ*_C_ (mult.) *^b^*	*δ*_H_ (*J* in Hz) *^a^*	*δ*_C_ (mult.) *^b^*
1		47.9 (qC)		47.4 (qC)		47.5 (qC)
2	4.89 d (8.8) *^c^*	74.7 (CH)	4.88 d (8.4)	74.2 (CH)	5.06 d (8.0)	74.4 (CH)
3	1.89 m	39.8 (CH_2_)	2.02 m	39.3 (CH_2_)	2.03 m	40.2 (CH)
	3.30 dd (15.6, 12.2)		2.81 dd (15.2, 12.0)		2.88 dd (15.2, 12.0)	
4	4.10 dd (12.2, 4.6)	71.2 (CH)	4.15 dd (12.0, 5.2)	69.2 (CH)	4.14 dd (12.0, 4.8)	70.8 (CH)
5		145.6 (qC)		145.3 (qC)		145.8 (qC)
6	5.27 d (8.4)	121.8 (CH)	5.53 d (8.8)	125.4 (CH)	5.35 d (8.8)	122.3 (CH)
7	6.18 d (8.4)	74.9 (CH)	5.81 d (8.8)	73.3 (CH)	5.78 d (8.8)	73.7 (CH)
8		72.0 (qC)		70.5 (qC)		70.4 (qC)
9	4.65 d (3.6)	66.7 (CH)	5.79 d (3.6)	67.0 (CH)	5.83 br s	67.2 (CH)
10	1.91 m	49.3 (CH)	2.10 m	49.0 (CH)	2.39 s	45.3 (CH)
11		78.4 (qC)		78.1 (qC)		73.8 (qC)
12	3.67 dd (12.2, 4.6)	73.7 (CH)	3.72 dd (12.4, 4.0)	73.3 (CH)	4.80 m	74.0 (CH)
13	2.02 td (12.2, 2.0)	30.2 (CH_2_)	2.04 td (12.4, 2.0)	30.2 (CH_2_)	2.00 m	25.8 (CH_2_)
	1.72 m		1.68 m		2.21 m	
14	4.77 dd (2.2, 2.0)	75.1 (CH)	4.83 dd (2.4, 2.0)	74.7 (CH)	4.70 br s	73.4 (CH)
15	1.33 s	14.2 (CH_3_)	1.26 s	14.5 (CH_3_)	1.26 s	14.4 (CH_3_)
16	2.03 d (1.2)	25.4 (CH_3_)	4.61 d (10.0)	68.3 (CH_2_)	2.10 d (1.2)	25.3 (CH_3_)
			4.75 dd (10.0, 1.6)			
17		65.2 (qC)		66.3 (qC)		66.1 (qC)
18	1.66 s	9.2 (CH_3_)	1.79 s	10.3 (CH_3_)	1.77 s	10.3 (CH_3_)
19		173.1 (qC)		170.2 (qC)		170.5 (qC)
20	1.32 s	17.4 (CH_3_)	1.16 s	17.0 (CH_3_)	1.25 s	23.2 (CH_3_)
OAc	1.99 s	21.5 (CH_3_)	2.00 s	21.4 (CH_3_)	1.98 s	21.4 (CH_3_)
	2.00 s	21.3 (CH_3_)	2.26 s	21.3 (CH_3_)	1.97 s	21.2 (CH_3_)
		170.4 (qC)	1.98 s	21.1 (CH_3_)	2.25 s	21.4 (CH_3_)
		170.4 (qC)	2.10 s	21.0 (CH_3_)		170.2 (qC)
				170.4 (qC)		170.1 (qC)
				168.3 (qC)		168.3 (qC)
				170.6 (qC)		
				171.5 (qC)		
*n*-Pr(CO)O					0.97 t (7.2)	13.6 (CH_3_)
					1.65 m	18.3 (CH_2_)
					2.37 m	36.3 (CH_2_)
						172.5 (qC)

*^a^* 400 MHz in CDCl_3_ (assigned by COSY, HSQC, and HMBC experiments); *^b^* 100 MHz in CDCl_3_ (assigned by DEPT, COSY, HSQC, and HMBC experiments); *^c^* *J* values (Hz) in parentheses.

The coupling information in the ^1^H–^1^H COSY experiment of **2** enabled the identification of the C-2/3/4, C-6/7, C-6/16 (by allylic coupling), C-9/10, and C-12/13/14 units. From these data, together with the results of an HMBC experiment of **2**, the molecular framework of **2** could be further established. The HMBC correlations also revealed that the acetate groups are attached at C-2, C-9, C-14, and C-16; thus, the remaining hydroxy groups should be positioned at C-4, C-11, and C-12. The relative stereochemistry of **2** was elucidated from the NOE interactions observed in an NOESY experiment (Figure 4) and the configurations of all chiral centers of **2** were confirmed as being the same as those of **1** by comparison of the proton chemical shifts, coupling constants, and NOE correlations.

**Figure 5:**
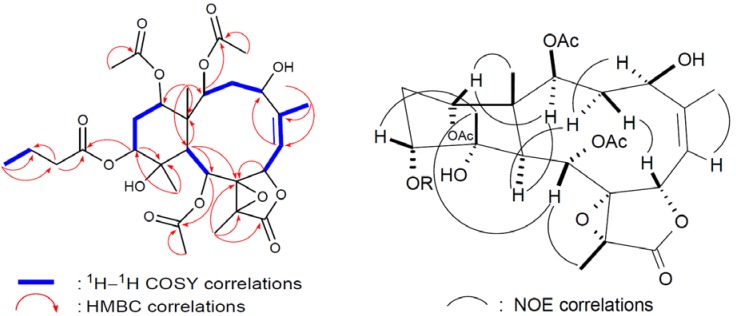
2D NMR correlations of compound **3**.

Briacavatolide C (**3**) was isolated as a white solid and had the molecular formula C_30_H_42_O_13_, as determined by HRFABMS. The presence of hydroxyl, γ-lactone, and ester groups were evident from IR absorptions at 3500, 1779, and 1738 cm^−1^, respectively. ^1^H and ^13^C NMR spectral data (Table 1) revealed that **3** contains a trisubstituted double bond. The gross structure of **3 **and all of the ^1^H and ^13^C chemical shifts associated with the molecule were determined by a series of 2D NMR experiments (Figure 5). In the HMBC spectrum of **3**, the *n*-butyrate positioned at C-12 was confirmed from the connectivity between H-12 (*δ*_H_ 4.80) with the carbonyl carbon (*δ*_C_ 172.5) of the *n*-butyryloxyl group. Furthermore, the HMBC correlations also revealed that three acetates were attached to C-2, C-9, and C-14. These data, together with the other ^1^H-^13^C long-range correlations (Table 1), unambiguously established the molecular framework of **3**. The relative configurations of **3 **were identical to those of **1** except that of C-12. H-12 was found to exhibit NOE correlations (Figure 5) with H-13α, H-13β, and H_3_-20, but not with H-10, revealing the β-orientation of H-12.

Briacavatolides A–C (**1**–**3**) and known compounds briaexcavatolide U (**4**) and briaexcavatin L (**5**) were evaluated for cytotoxicity against P-388 (mouse lymphocytic leukemia), HT-29 (human colon adenocarcinoma), and A-549 (human lung epithelial carcinoma) tumor cells. No cytotoxic activity was detected for any of the isolated compounds against all cell lines at 100 μM. The compounds were also examined for antiviral activity against human cytomegalovirus (HCMV) using a human embryonic lung (HEL) cell line. Briacavatolide C (**3**) was found to have anti-HCMV activity with an IC_50_ of 18 μM.

## 3. Experimental Section

### 3.1. General Experimental Procedures

Optical rotations were determined with a JASCO P1020 digital polarimeter. UV and IR spectra were obtained on JASCO V-650 and JASCO FT/IR-4100 spectrophotometers, respectively. NMR spectra were recorded on a Varian MR 400 NMR spectrometer at 400 MHz for ^1^H and 100 MHz for ^13^C, respectively. ^1^H NMR chemical shifts are expressed in *δ* values referring to the solvent peak *δ*_H_ 7.27 for CDCl_3_, and coupling constants are expressed in Hz. ^13^C NMR chemical shifts are expressed in *δ* (ppm) referring to the solvent peak *δ*_C_ 77.0 for CDCl_3_. MS were recorded by a Bruker APEX II mass spectrometer. Silica gel 60 (Merck, Germany, 230–400 mesh) and LiChroprep RP-18 (Merck, 40–63 μm) were used for column chromatography. Precoated silica gel plates (Merck, Kieselgel 60 F_254_, 0.25 mm) and precoated RP-18 F_254s_ plates (Merck) were used for thin-layer chromatography (TLC) analysis. High-performance liquid chromatography (HPLC) was carried out using a Hitachi L-7100 pump equipped with a Hitachi L-7400 UV detector at 220 nm together with a semi-preparative reversed-phase column (Merck, Hibar LiChrospher RP-18e, 5 μm, 250 × 25 mm).

### 3.2. Biological Material

The octocoral *B. excavatum* was collected by hand using scuba at Orchid Island off Taiwan, in July 2008 at a depth of 12 m and stored in a freezer until extraction. The voucher specimen (LY-05) was identified by Prof. Chang-Feng Dai, National Taiwan University and deposited at the Department of Marine Biotechnology and Resources, National Sun Yat-sen University, Taiwan.

### 3.3. Extraction and Isolation

A specimen of octocoral *B. excavatum* (1.5 kg) was minced and extracted with acetone (2 L × 5) at room temperature. The combined acetone extracts was then partitioned between H_2_O and EtOAc. The resulting EtOAc extract (30.5 g) was subjected to gravity silica gel 60 column chromatography (Si 60 CC) using *n*-hexane and *n*-hexane/EtOAc of increasing polarity, to give 20 fractions. Fraction 12 (3.0 g), eluted with *n*-hexane/EtOAc (1:10), was further subjected to Si 60 CC (*n*-hexane/EtOAc, 10:1) to give 9 subfractions. A subfraction 12-5 (360 mg) was separated by a RP-18 flash column (MeOH/H_2_O, 50:50 to 100% MeOH) to give 6 fractions. In turn, a subfraction 12-5-2, eluted with MeOH/H_2_O (65:35), was further purified by RP-18 HPLC (MeOH/H_2_O, 60:40) to afford **3 **(2.0 mg). A subfraction 12-6 (380 mg) was separated by RP-18 HPLC (MeOH/H_2_O, 60:40) to give **4 **(1.0 mg). Similarly, fraction 13 (0.63 g), eluted with EtOAc/MeOH (90:1), was further subjected to Si 60 CC (*n*-hexane/EtOAc, 10:1 to EtOAc) to give 9 subfractions. A subfraction 13-8 (128 mg), was separated by a RP-18 flash column (MeOH/H_2_O, 40:60 to 100% MeOH) to give 7 fractions. The subfraction 13-8-3, eluted with MeOH/H_2_O (50:50), was purified by RP-18 HPLC (MeOH/H_2_O, 47:53) to afford **1 **(2.0 mg) and **5 **(3.0 mg). Likewise, the subfraction 13-9 (62 mg), was separated by a RP-18 flash column (MeOH/H_2_O, 40:60 to 100% MeOH) to give 7 fractions. The fraction 14 (0.14 g), eluted with EtOAc/MeOH (70:1), was further subjected to a RP-18 flash column (MeOH/H_2_O, 40:60 to 100% MeOH) to give 7 fractions. The subfraction 14-1, eluted with MeOH/H_2_O (40:60), was purified by RP-18 HPLC (MeOH/H_2_O, 37:63) to afford **2 **(3.0 mg).

Briacavatolide A (**1**): White amorphous powder; [α]_D_^25^ +83.2 (*c* 0.1, CHCl_3_); IR (neat) ν_max_ 3364, 2921, 1764, 1712, 1374, 1264, 1095 cm^−1^; ^1^H NMR (CDCl_3_, 400 MHz) and ^13^C NMR (CDCl_3_, 100 MHz) data in Table 1; HRESIMS *m/z* 521.1998 [M + Na]^+^ (calcd for C_24_H_34_O_11_Na, 521.1999).

Briacavatolide B(**2**): White amorphous powder; [α]_D_^25^−57.8 (*c* 0.1, CHCl_3_); IR (neat) ν_max_ 3448, 2938, 1777, 1734, 1373, 1258, 1083 cm^−1^; ^1^H NMR (CDCl_3_, 400 MHz) and ^13^C NMR (CDCl_3_, 100 MHz) data in Table 1; HRESIMS *m/z* 621.2163 [M + Na]^+^ (calcd for C_28_H_38_O_14_Na, 621.2159).

Briacavatolide C(**3**): White amorphous powder; [α]_D_^25^ +25.5 (*c* 0.1, CHCl_3_); IR (neat) ν_max_ 3500, 2965, 1779, 1738, 1371, 1256, 1022 cm^−1^; ^1^H NMR (CDCl_3_, 400 MHz) and ^13^C NMR (CDCl_3_, 100 MHz) data in Table 1; HRESIMS *m/z* 633.2527 [M + Na]^+^ (calcd for C_30_H_42_O_13_Na, 633.2523).

### 3.4. Cytotoxicity Assay

Cytotoxicity was determined on P-388 (mouse lymphocytic leukemia), HT-29 (human colon adenocarcinoma), and A-549 (human lung epithelial carcinoma) tumor cells using a modification of the MTT colorimetric method according to a previously described procedure [6,7]. The provision of the P-388 cell line was supported by J.M. Pezzuto, formerly of the Department of Medicinal Chemistry and Pharmacognosy, University of Illinois at Chicago. HT-29 and A-549 cell lines were purchased from the American Type Culture Collection. To measure the cytotoxic activities of tested compounds, five concentrations with three replications were performed on each cell line. Mithramycin was used as a positive control.

### 3.5. Anti-HCMV Assay

To determine the effects of natural products upon HCMV cytopathic effect (CPE), confluent human embryonic lung (HEL) cells grown in 24-well plates were incubated for 1 h in the presence or absence of various concentrations of tested natural products with three replications. Ganciclovir was used as a positive control. Then, cells were infected with HCMV at an input of 1000 pfu (plaque forming units) per well of a 24-well dish. Antiviral activity was expressed as IC_50_ (50% inhibitory concentration), or compound concentration required to reduce virus induced CPE by 50% after 7 days as compared with the untreated control. To monitor the cell growth upon treating with natural products, an MTT-colorimetric assay was employed [8].

## 4. Conclusion

The first investigation of octocoral *B. excavatum* collected at Orchid Island has led to the isolation of three new briarane-type diterpenoids, briacavatolides A–C (**1**–**3**) as well as two known briaranes, briaexcavatolide U (**4**) and briaexcavatin L (**5**). Briacavatolide C (**3**)containing *n*-butyryloxyl groups was found to show anti-HCMV activity with an IC_50_ of 18 μM. 
